# Causal relationship between type 2 diabetes and common respiratory system diseases: a two-sample Mendelian randomization analysis

**DOI:** 10.3389/fmed.2024.1332664

**Published:** 2024-07-18

**Authors:** Jie Chen, Xiaofeng Zhang, Gengyun Sun

**Affiliations:** ^1^Department of Respiratory and Critical Care Medicine, The First Affiliated Hospital of Anhui Medical University, Hefei, China; ^2^Department of Respiratory and Critical Care Medicine, The Third Affiliated Hospital of Anhui Medical University, Hefei, China; ^3^General Medicine Department, The Second Affiliated Hospital of Anhui Medical University, Hefei, China

**Keywords:** Mendelian randomization analysis, type 2 diabetes, chronic obstructive pulmonary disease, bronchial asthma, lung cancer, interstitial lung disease, pulmonary tuberculosis

## Abstract

**Background:**

Type 2 diabetes (T2D) frequently co-occurs with respiratory system diseases such as chronic obstructive pulmonary disease (COPD), bronchial asthma, lung cancer, interstitial lung disease, and pulmonary tuberculosis. Although a potential association is noted between these conditions, the available research is limited.

**Objective:**

To investigate the causal relationship between patients with T2D and respiratory system diseases using two-sample Mendelian randomization analysis.

**Methods:**

Causal relationships were inferred using a two-sample Mendelian randomization (MR) analysis based on publicly available genome-wide association studies. We employed the variance inverse-weighted method as the primary analytical approach based on three key assumptions underlying MR analysis. To bolster the robustness and reliability of our results, we utilized MR Egger’s intercept test to detect potential pleiotropy, Cochran’s Q test to assess heterogeneity, funnel plots to visualize potential bias, and “leave-one-out” sensitivity analysis to ensure that our findings were not unduly influenced by any single genetic variant.

**Result:**

The inverse variance weighted (IVW) analysis indicated a causal relationship between T2D and COPD [Odds Ratio (OR) = 0.87; 95% Confidence Interval (CI) = 0.82–0.96; *p* < 0.05]. No significant heterogeneity or pleiotropy were observed through their respective tests (*p* > 0.05), and the statistical power calculations indicated that the results were reliable. The IVW analysis showed a negative causal relationship between T2D and bronchial asthma [OR = 0.85; 95% CI = 0.81–0.89; *p* < 0.05]. However, the IVW under the random-effects model indicated heterogeneity (*p* < 0.05), suggesting instability in the results and requiring cautious interpretation. The study found a positive causal relationship between T2D and pulmonary tuberculosis (OR = 1.24, 95% CI = 1.05–1.45, *p* < 0.05). However, they exhibited pleiotropy (*p* < 0.05), indicating their instability. No correlation between T2D and interstitial lung disease or lung cancer was observed.

**Conclusion:**

T2D is negatively associated with COPD, suggesting that T2D may reduce the risk of developing COPD. A negative causal relationship between T2D and bronchial asthma has been observed, but the results exhibit heterogeneity. There is a positive causal relationship between T2D and pulmonary tuberculosis, yet the findings suggest the presence of pleiotropy. No significant causal relationship between T2D and lung cancer or interstitial lung disease was observed.

## 1 Introduction

Respiratory system diseases, including chronic obstructive pulmonary disease (COPD), bronchial asthma, lung cancer, interstitial lung disease, and pulmonary tuberculosis (PTB), present notable global health challenges. These conditions collectively contribute to substantial morbidity and mortality, resulting in considerable socio-economic impacts (1, 2). Diabetes is a common chronic metabolic disease that had a global prevalence of 9.3% in 2019, which is projected to increase to 10.9% by 2045 (3). Prolonged hyperglycemia can lead to a range of serious complications, including cardiovascular diseases, nephropathy, retinopathy, and neuropathy (4). These complications substantially increase the disease burden and the risk of premature death in patients, placing a tremendous economic strain on healthcare systems (5).

Respiratory system diseases are also prevalent and are typically influenced by multiple factors, including smoking, indoor and outdoor air pollution, allergens, occupational factors, and genetics (1). Type 2 diabetes (T2D) accounts for over 95% of diabetes cases and frequently coexists with respiratory system diseases, thus suggesting a potential association between them. For instance, a cross-sectional study found that after adjusting for confounding factors, patients with T2D had an increased risk of developing COPD [Odds Ratio (OR) = 1.45; 95% Confidence Interval (CI) = 1.23–1.71; *p* < 0.05] and bronchial asthma (OR = 1.38; 95% CI = 1.24–1.53; *p* < 0.05) (6). Additionally, author study showed that patients with T2D are more susceptible to PTB (7). Furthermore, two prospective cohort studies in the United States have found that T2D is associated with a higher risk of lung cancer (8). However, a prospective cohort study from Shanghai, China, observed no association between T2D and lung cancer risk in men [HR (Hazard Ratio) = 0.87, 95% CI 0.62–1.21] or women (HR = 0.92; 95% CI 0.69–1.24) (9). The limited number of studies and inconsistent results may be due to methodological differences, insufficient sample sizes, varying interpretations of the results, or inadequate control of confounding factors.

Using Mendelian randomization (MR) method to investigate this relationship offers a unique opportunity to elucidate potential causal links. MR is often referred to as “nature’s randomized double-blind trial.” This approach offers three key advantages. First, genetic information serves as an instrumental variable that is less susceptible to confounding factors. Secondly, MR is less affected by reverse causality. Finally, MR results can provide insights into the directionality of the relationship between exposure and outcome, extending beyond mere associations.

Considering the current gaps and uncertainties in the existing literature, this study aimed to employ MR techniques to systematically explore the potential connection between T2D and respiratory system diseases.

## 2 Materials and methods

### 2.1 Study design and data source

Mendelian randomization (MR) techniques were used to assess the causal relationship between type 2 diabetes (T2D) and common respiratory system diseases. The instrumental variable was T2D, and the outcome variables included chronic obstructive pulmonary disease (COPD), bronchial asthma, lung cancer, interstitial lung disease, and pulmonary tuberculosis (PTB).

Exposure-and outcome-related datasets were derived from a genome-wide association study (GWAS), as indicated in Table 1. All participants were recruited under the Japanese Biobank Project (BBJ) (10, 11). Diagnoses of all study participants’ diseases were made in collaboration with hospital physicians. Written informed consent was obtained from all participants with approval from the RIKEN Center for Integrative Medical Sciences and the Ethics Committee of the University of Tokyo School of Medicine.

**Table 1:** Study overview: sample size and pooled GWAS for each outcome of interest.

	Consortia	Year	Sample size (case/control)	Number of SNPs	Population	Dataset
Exposure						
T2D	BBJ	2019	210,865 (40,250/170615)	8,885,694	East Asian	bbj-a-153
Outcome						
COPD	BBJ	2019	204,907 (3315/201592)	8,885,538	East Asian	bbj-a-103
Asthma	BBJ	2019	209,808 (8216/201592)	8,885,667	East Asian	bbj-a-88
Interstitial lung disease	BBJ	2019	424,100 (212,453/211647)	8,885,805	East Asian	bbj-a-127
Lung cancer	BBJ	2019	420,856 (212,453/208403)	8,885,805	East Asian	bbj-a-133
Pulmonary tuberculosis	BBJ	2019	424,357 (212,453/211904)	8,885,805	East Asian	bbj-a-149

T2D, type 2 diabetes. COPD, chronic obstructive plumonary disease. BBJ, BioBank Japan.

### 2.2 The selection and validation of SNPs

**Figure 1:**
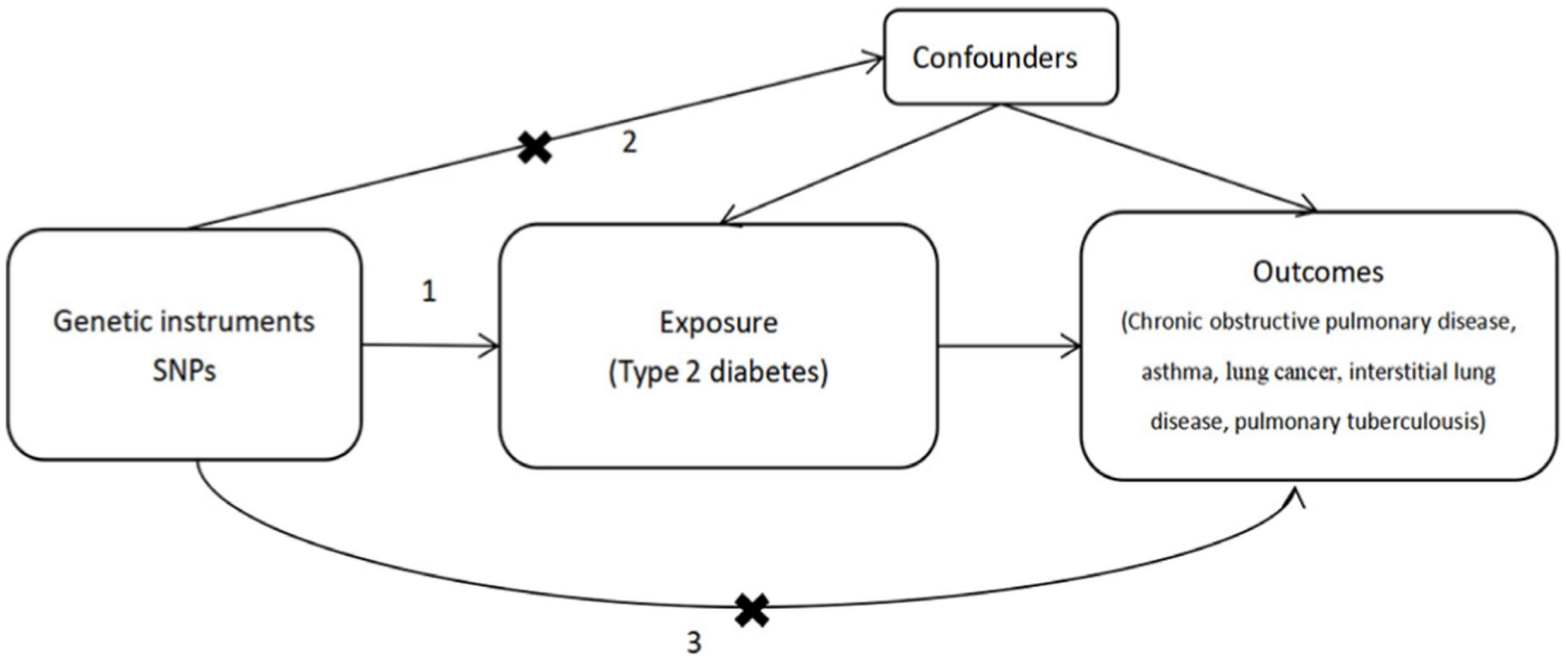
Mendelian randomization analysis must adhere to three key assumptions: 1. SNPs are stongly associated with the exposure (type 2 disbetes), 2. SNPs do not influence the outcome through other confounders, 3. SPNs only affect the outcomes through the exposure.

MR analysis necessitates the fulfillment of three crucial assumptions (Figure 1). First, SNPs must exhibit strong associations with the exposure variable (Assumption 1). Second, SNPs should not influence the outcome through other confounding factors (Assumption 2). Third, they should solely impact the outcome through the exposure variable (Assumption 3). To adhere to these three key assumptions, we selected SNPs that were significantly associated with T2D at the whole-genome level (using a filtering threshold of *p* < 5 × 10^−8^).

To ensure independence among the SNPs, we eliminated linkage disequilibrium (LD) by requiring *R*^2^ < 0.001 and a window size of 10,000 kb. To uphold Assumption 2, which posits that genetic variation is unrelated to potential confounders, we conducted a query in the Phenoscanner database to confirm that the included SNPs were unrelated to known confounding factors (smoking, indoor or outdoor air pollution, allergens, HIV infection, occupational and genetic factors, lung function (FEV1, FEV1/FVC), eosinophils, socioeconomic status, and immunosuppression). Finally, we computed F-test values to validate the strength of the individual SNPs. When the F-statistics were greater than 10, the SNPs were considered powerful enough to mitigate the influence of potential bias.

### 2.3 Mendelian randomization analysis

We employed the inverse variance weighted (IVW) method to conduct a two-sample MR analysis to explore the causal relationships between these variables. Additionally, we utilized the MR-Egger and weighted median approaches for further analysis.

Sensitivity analyses were conducted from multiple perspectives to assess the robustness of results. First, we performed a heterogeneity assessment using the Cochran Q test, with results indicating heterogeneity at *p* < 0.05. In such cases, we analyzed the data using the IVW method with a random-effects model. Otherwise, we utilized the IVW and MR-Egger methods under a fixed-effects model. Second, we applied leave-one-out sensitivity analysis and funnel plot techniques to investigate potential heterogeneity in the results. Finally, we conducted a pleiotropy assessment using MR-Egger regression, with significance set at *p* < 0.05, indicating the presence of pleiotropy. Beyond sensitivity analysis, we employed funnel, leave-one-out, scatter, and forest plots to visualize the results.

All statistical analyses were performed using two-tailed Student’s t-tests. Statistical significance was set at *p* < 0.05 indicated statistical significance. All statistical analyses were performed using the “TwoSampleMR” package within R version 4.3.1.

### 2.4 Statistical power

The statistical power of the MR analysis was assessed using the online tool, mRND (12) to validate the reliability of the results.

## 3 Results

A total of 92 SNPs met the three fundamental criteria of Mendelian randomization (MR) and achieved genome-wide significance. All F-statistics were greater than 10 (Supplementary Table S1).

### 3.1 The causality between type 2 diabetes and chronic obstructive pulmonary disease

**Figure 2:**
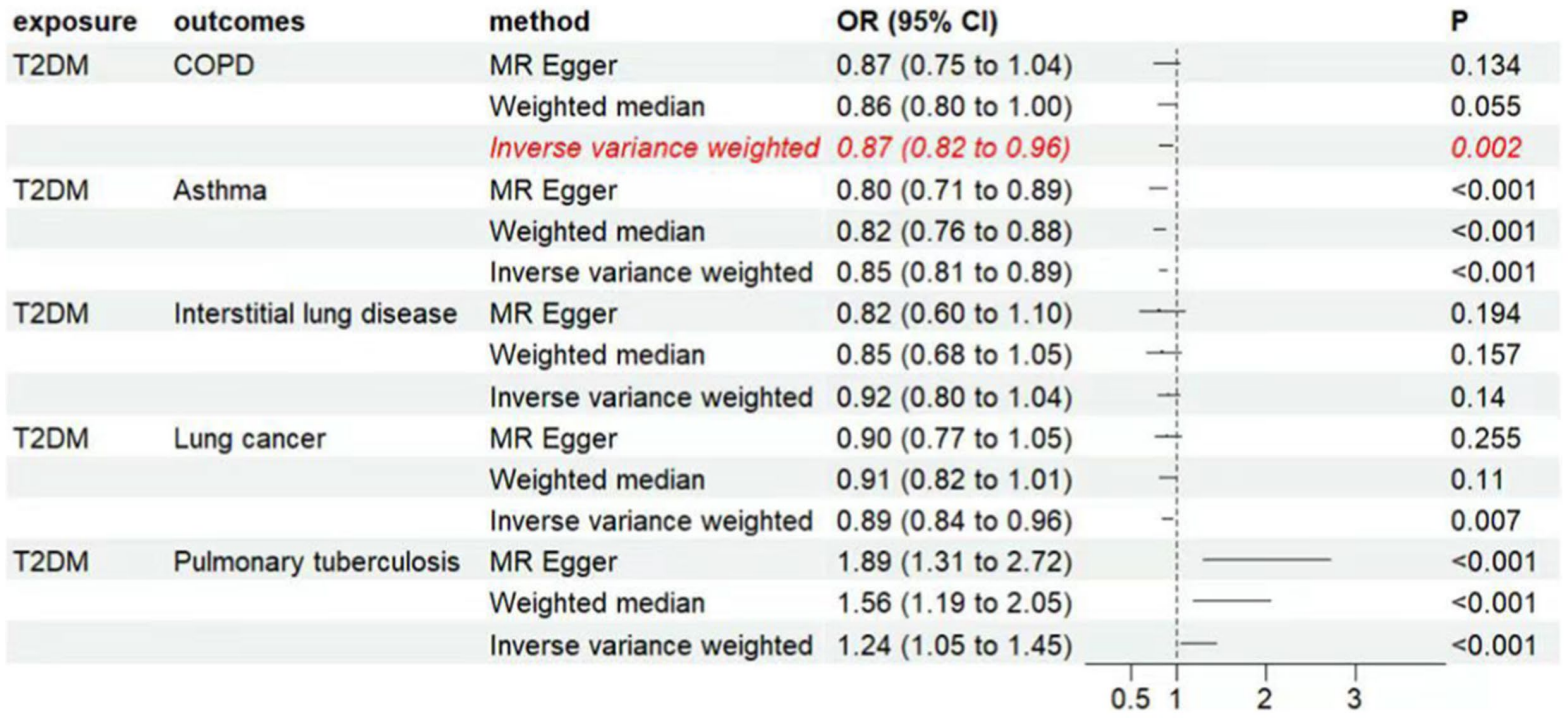
Associations of genetically predicted type 2 diabetes with respiratory system diseases. CI, condidence interval; OR, odds ratio; T2DM, type 2 diabetes mellitus, COPD, chronic obstructive pulmonary disease.

**Figure 3:**
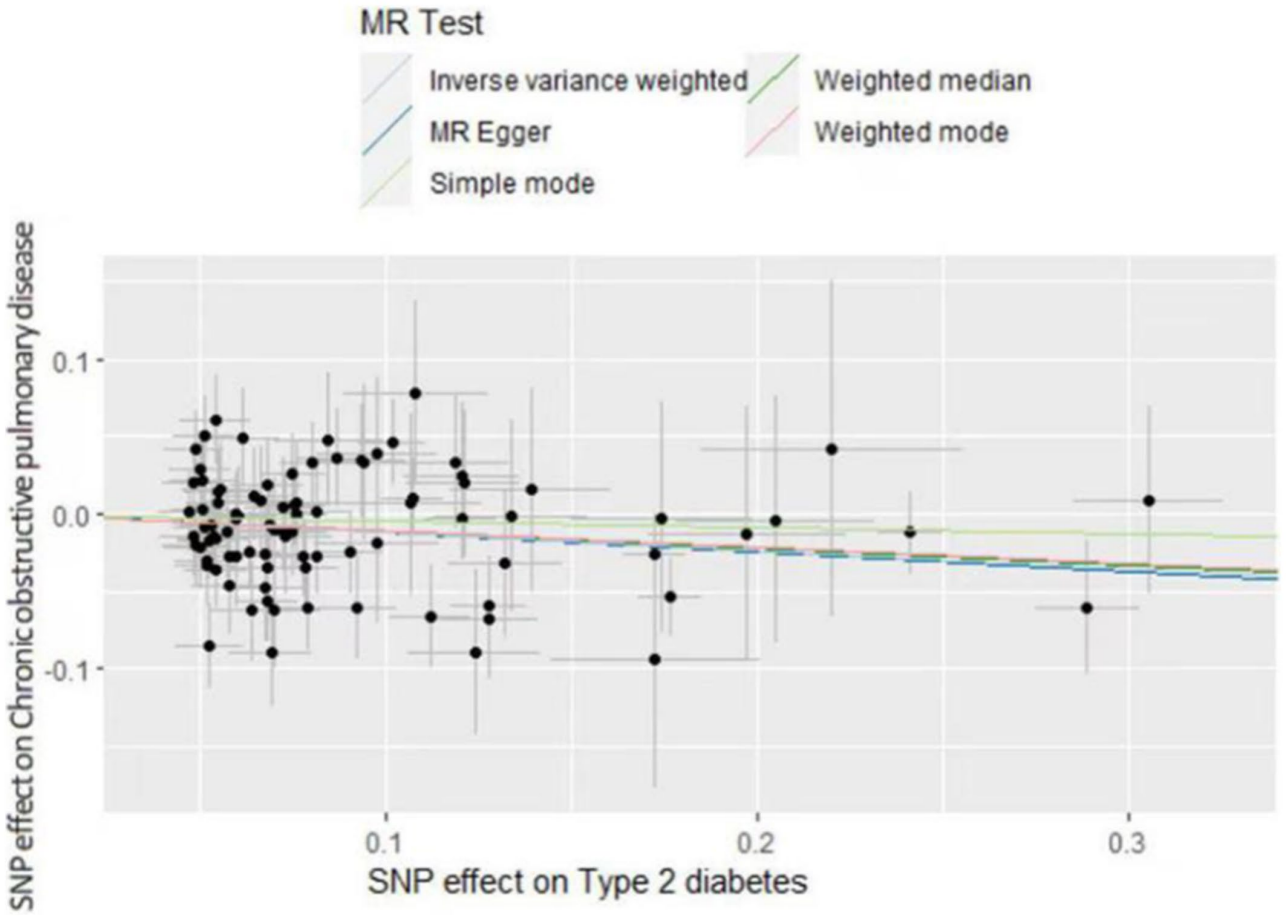
Genetic associations between type 2 diabetes and chronic obstructive disease.

The inverse variance weighted (IVW) analysis results indicate a causal relationship between T2D and COPD (OR = 0.87, 95%CI 0.82–0.96, *p* < 0.05). The weighted-median and MR-Egger conclusions were inconsistent with the IVW results (*p* > 0.05) (Figure 2). Tests for heterogeneity and pleiotropy suggested no significant heterogeneity or pleiotropy (*p* > 0.05) (Table 2). The funnel plot did not show any apparent bias (Supplementary Figure S1) and the results remained consistent when individual SNPs were progressively removed from the leave-one-out analysis (Supplementary Figure S2). The scatter and forest plots also aligned with these results (Figure 3; Supplementary Figure S4).

**Table 2:** Heterogeneity and pleiotropy analysis for the effect of T2D on the risk of respiratory system diseases.

Exposure	Outcomes	Methods	Heterogeneity		Pleiotropy	
			Q	*p* value	Egger_intercept	*p* value
T2D	COPD	MR Egger	99.4	0.211^*^	0.0006	0.929
		IVW	99.4	0.233^*^		
T2D	Asthma	MR Egger	–	–	0.0070	0.195
		IVW	–	1.48 × 10^–9#^		
T2D	Pulmonary tuberculosis	MR Egger	89.5	0.464^*^	−0.0473	0.007
		IVW	97.4	0.280^*^		

*p-values under a fixed effects model; #,p-values under a random effects model.

### 3.2 The causality between T2D and asthma, lung cancer, interstitial lung disease, and pulmonary tuberculosis

The IVW analysis results indicate a negative causal relationship between T2D and bronchial asthma (OR = 0.85, 95% CI 0.81–0.89, *p* < 0.001; Figure 2). This conclusion is consistent with those of the weighted-median and MR-Egger methods. However, both the fixed-effects model and IVW under the random-effects model suggested the presence of heterogeneity (*p* < 0.05; Table 2). T2D was found to have a positive causal relationship with PTB (Figure 2), but exhibited pleiotropy (*p* < 0.05; Table 2). There was no correlation between T2D and interstitial lung disease or lung cancer (Figure 2). Additional funnel, leave-one-out, scatter, and forest plots are shown in Supplementary Figures S1–S4.

### 3.3 Statistical power

The relevant data were input into the mRND online tool, yielding a statistical power of 1, indicating the high reliability of the MR analysis results for T2D and respiratory system diseases.

## 4 Discussion

This study identified a negative causal association between type 2 diabetes (T2D) and bronchial asthma, but with significant heterogeneity. This may be because of several reasons. First, conducting a stratified analysis of the data could potentially identify characteristic populations. Second, chronic obstructive pulmonary disease (COPD) is generally characterized by neutrophilic inflammation, whereas asthma is characterized by eosinophilic inflammation (13). Diabetes predominantly hinders neutrophil migration and phagocytosis (14), indicating a close association with COPD. Third, from an immunological perspective, persistently high blood sugar levels can negatively affect immune system function, reducing its responsiveness to infections and inflammation (15). Persistent immunosuppression manifests as sustained irreversible airflow limitation during airway inflammation, whereas asthma presents as reversible airflow limitation.

In relation to pulmonary tuberculosis (PTB), Clinical studies have demonstrated that patients with T2D are more susceptible to PTB infection (7, 16) and have a higher risk of mortality (17). This increased risk is likely due to the compromised immune function and reduced immune response associated with T2D (18). Some confounding factors such as low socioeconomic status, poor living conditions, and impaired immunity (19, 20), which are common risk factors for both T2D and PTB, were not excluded from these studies. Our Mendelian randomization (MR) study also identified a positive causal relationship between T2D and PTB, but showed pleiotropy, indicating unstable results. Notably, although we excluded SNPs related to socioeconomic status, living conditions, and immunity when selecting the exposure-related SNPs, there may still be some confounding factors or indirect influences through other intermediate phenotypes. Metformin is the first-line therapy for T2D and most of the diabetes patients might have taken metformin. Metformin has been shown to reduce the risk of many bacterial and viral infections including zika virus (21), dengue virus (21), Hepatitis C virus (22), *Streptococcus pneumoniae* (23) and PTB (24). The use of metformin in many of the patients with T2D might have modified and attenuated the positive association between T2D and PTB. This could be a contributing factor to the pleiotropy observed in our study. The evidence regarding the association between T2D and lung cancer is limited and inconsistent. A real-world study from Shanghai, China showed an increased risk of lung cancer in patients with T2D (25) and an epidemiological follow-up of a population-based cohort of diabetes patients of the Chinese ethnicity living in Taiwan over a 12-year period from 1995 to 2006 strongly supported an increased mortality from lung cancer in the diabetes patients while compared to the general population (26). However, an MR study involving European populations found no association between diabetes and lung cancer (27). Our finding of a lack of association between diabetes and lung cancer might be due to use of the databases not derived from the Chinese ethnicities. Research on the association between T2D and interstitial lung disease is limited. A MR study on T2D and idiopathic pulmonary fibrosis showed no significant association (28). Future large-scale prospective studies are warranted to further explore causality, considering various demographic characteristics (such as age, genetics, and lifestyle) through subgroup analyses in different populations.

More importantly, our results indicate that T2D may reduce the risk of developing COPD. A one-half standard deviation increase in genetically predicted T2D was associated with a decreased likelihood of COPD (OR = 0.87, 95% CI 0.82–0.96, *p* < 0.05), without heterogeneity or pleiotropy, suggesting more stable results. Clinical studies on the relationship between T2D and COPD are limited. Some studies have suggested an increased risk of future COPD among patients with T2D (29), a view that has relatively more support (30). However, the results of a few studies are consistent with those of this study. A retrospective case–control study matched 29,217 patients with T2D with controls in a 1:1 ratio and followed them for 8 years, showing a decreased risk of COPD among patients with T2D (HR 0.89, 95% CI 0.79–0.93) (31). It is noteworthy that in some East Asian study populations, T2D was associated with restrictive lung impairment, but not with obstructive lung impairment (32, 33). These findings suggest that T2D is not associated with an increased risk of COPD. We observed that reduced lung function precedes and significantly predicts the future development of T2D (34, 35), consistent with studies assessing the association between T2D and COPD, focusing on the timing of diagnosis, as in our study. There are several possible explanations for the decreased incidence of COPD associated with T2D.

Regarding common risk factors, substantial evidence indicates that smoking is a causative factor of COPD. Additionally, smoking also plays a role in the progression of T2D (36). Individuals diagnosed with T2D were more likely to receive smoking cessation advice from healthcare professionals than those without T2D. This leads to a reduced risk of COPD in this population. Secondly, T2D involves metabolic disturbances that can weaken the inflammatory response.

Secondly, T2D involves metabolic disturbances that can lead to weakened inflammatory responses. First, chronic hyperglycemia can compromise immune system function (15), diminishing the immune system’s ability to respond to infections and inflammation. For instance, high blood sugar levels can inhibit neutrophil migration and phagocytosis, and suppress superoxide production and microbial killing (14), which is detrimental to the anti-inflammatory action of white blood cells, such as macrophages and T cells. Second, high blood sugar levels lead to increased oxidative stress, resulting in excessive production of reactive oxygen species within the body. Reactive oxygen species can damage cells and tissues, and stimulate inflammatory responses. Prolonged oxidative stress can disrupt the immune system, making it difficult to cope with inflammation (37). Finally, a high blood sugar level can impair the function of endothelial cells (inner lining of blood vessels), leading to vascular inflammation (38). This affects tne adhesion and migration of immune cells, thereby weakening the inflammatory response of the immune system.

Third, metformin is a first-line medication for patients with T2D. It inhibits gluconeogenesis by activating AMP-activated protein kinase, which is a crucial mechanism in diabetes and related metabolic disorders (39). Increasing evidence suggests that metformin can benefit patients with COPD. Animal studies suggested that metformin protects against cigarette smoke-induced lung inflammation and emphysema. Compared to participants not treated with metformin, those receiving metformin therapy showed a slower progression in the percentage of emphysema (adjusted difference-in-difference of −0.92%; 95%CI, −1.7 to −0.14%) and experienced an attenuation of the decrease in lung density decrease (adjusted difference-in-difference of 2.2 g/L; 95% CI, 0.43 to 4.0 g/L) at 5 years (39). Observational studies have revealed the preventive effects of metformin on COPD in T2D patients, especially when metformin use exceeds 2 years. This beneficial effect demonstrated a dose-dependent trend, and sensitivity analyses consistently supported this conclusion (40). Moreover, metformin can reduce the risk of acute exacerbation (41) and mortality (42, 43) in COPD. Pioglitazone primarily increases the sensitivity of the body to insulin. In a mouse model of emphysema, adipose-derived stem cells (ASCs) pretreated with pioglitazone showed a more effective therapeutic effect than ASCs without pretreatment (44). Retrospective cohort studies have shown that pioglitazone can significantly reduce the risk of COPD (HR = 0.778, 95% CI 0.667–0.908, *p* < 0.05), and this effect is more pronounced when used for >11 months (45).

Finally, the MR analysis typically utilizes genetic variations to simulate causal relationships. It is possible that certain unknown genetic factors reduce the risk of COPD in individuals with T2D. We know that there is a strong association between smoking and COPD. In an Asian dataset, some SNPs associated with T2D, such as rs10906115, rs459193, rs4607103, and rs4607517, exhibited significant interactions with smoking (46). In particular, the polymorphism rs5015480 in HHEX has been reported to be associated with serum glucose levels in multiple East Asian datasets (46–49), and interacts significantly with smoking (46, 47). This warrants further exploration and is an important direction for future research.

Our study has some limitations. First, although we excluded SNPs known to be associated with confounding factors such as smoking, lung function, environmental pollution, allergens, and occupational factors during SNP selection, we did not exclude SNPs related to antidiabetic drugs, which may have influenced the interpretation of our results. Studies have shown associations between medications such as metformin, pioglitazone, and insulin and respiratory system diseases, but the conclusions vary. For instance, some studies found no association between antidiabetic drugs and the risk of lung cancer (50), while others suggested a possible increased risk of insulin use (51), decreased risk with pioglitazone (52), and no significant association with lung cancer for pioglitazone (53). Data on the associations between insulin, pioglitazone, and PTB or interstitial lung diseases are limited. Currently, specific SNP information related to antidiabetic drugs is not available in the PhenoScanner database. Therefore, while excluding all SNPs related to antidiabetic drugs might be an option, it could reduce the statistical power, making it difficult to detect smaller effects. Additionally, the drug effects predicted based on genetics may not align with the actual treatment outcomes, as clinical drug use involves specific doses, durations, and timings, which our study could not capture regarding exposure to antidiabetic drugs during specific life stages. Thus, we hope that future studies will integrate clinical medication data for a more comprehensive investigation.

Regarding other limitations, first, since we used summary-level data, we were unable to conduct subgroup analyses, which limited our ability to explore causal relationships among different subgroups (e.g., age, sex, or diabetes severity). Second, there was sample selection bias. The use of the Japanese Biobank Project and differences in antidiabetic drugs used across different ethnicities and countries for T2D treatment may limit the applicability of our findings to the Chinese population. The study participants were of East Asian descent and may not represent the entire population. Given this, we extracted recent European datasets from GWAS for statistical analysis but did not find any causal association (Supplementary Table S2).

In conclusion, this study used MR analysis to reveal that T2D may serve as a protective factor against COPD. T2D is negatively associated with COPD, suggesting that T2D may reduce the risk of developing COPD. A negative causal relationship between T2D and bronchial asthma has been observed, but the results exhibit heterogeneity. There is a positive causal relationship between T2D and pulmonary tuberculosis, yet the findings suggest the presence of pleiotropy. No significant causal relationship between T2D and lung cancer or interstitial lung disease was observed. In the future, it would be worthwhile to conduct large-scale multicenter prospective randomized controlled studies to further validate these findings.

## Data availability statement

The original contributions presented in the study are included in the article/Supplementary material, further inquiries can be directed to the corresponding author.

## Ethics statement

The studies involving humans were approved by the Ethics Committee of the University of Tokyo School of Medicine. The studies were conducted in accordance with the local legislation and institutional requirements. The participants provided their written informed consent to participate in this study.

## Author contributions

JC: Writing – original draft, Data curation, Visualization. XZ: Software, Validation, Visualization, Writing – original draft. GS: Formal analysis, Resources, Writing – review & editing.
