# Supplementary material for: Causal relationship between type 2 diabetes and common respiratory system diseases: a two-sample Mendelian randomization analysis
Source: Front Med (Lausanne). 2024 Jul 18;11:1332664. doi: 10.3389/fmed.2024.1332664 (PMC11291206; doi:10.3389/fmed.2024.1332664)
Supplement: Supplementary file 1 [file Data_Sheet_1.PDF]

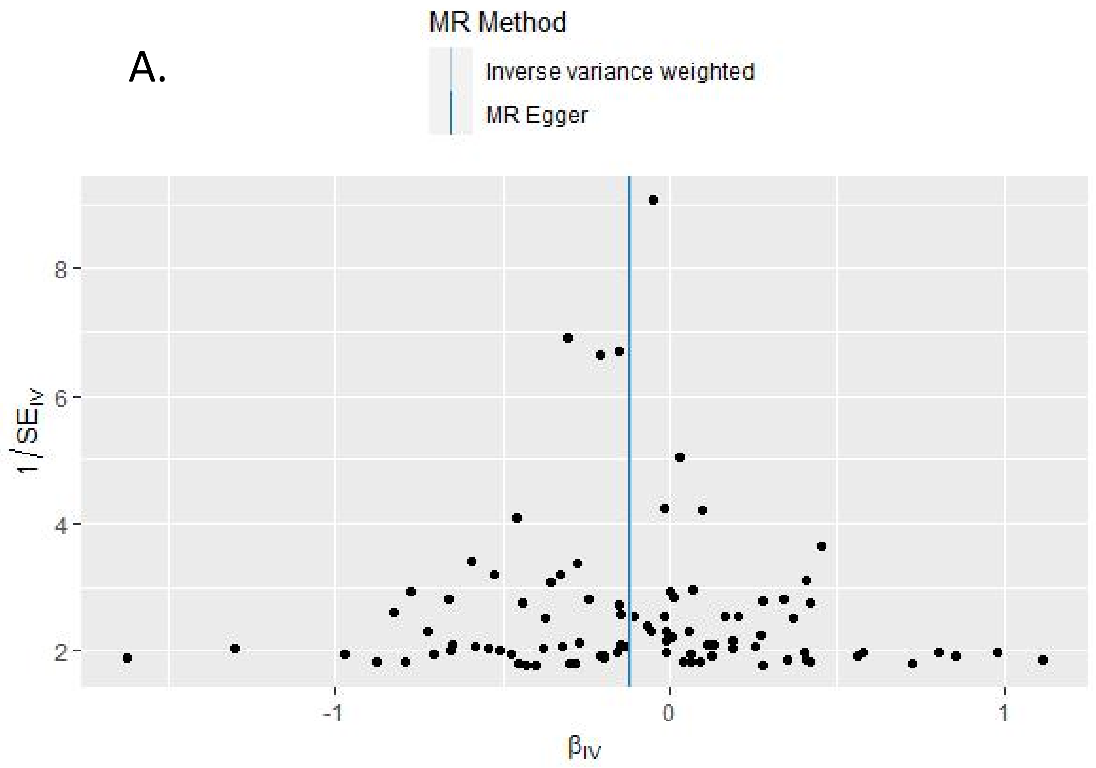

A. Funnel plot for the effect of type 2 diabetes on the risk of chronic obstructive pulmonary disease.

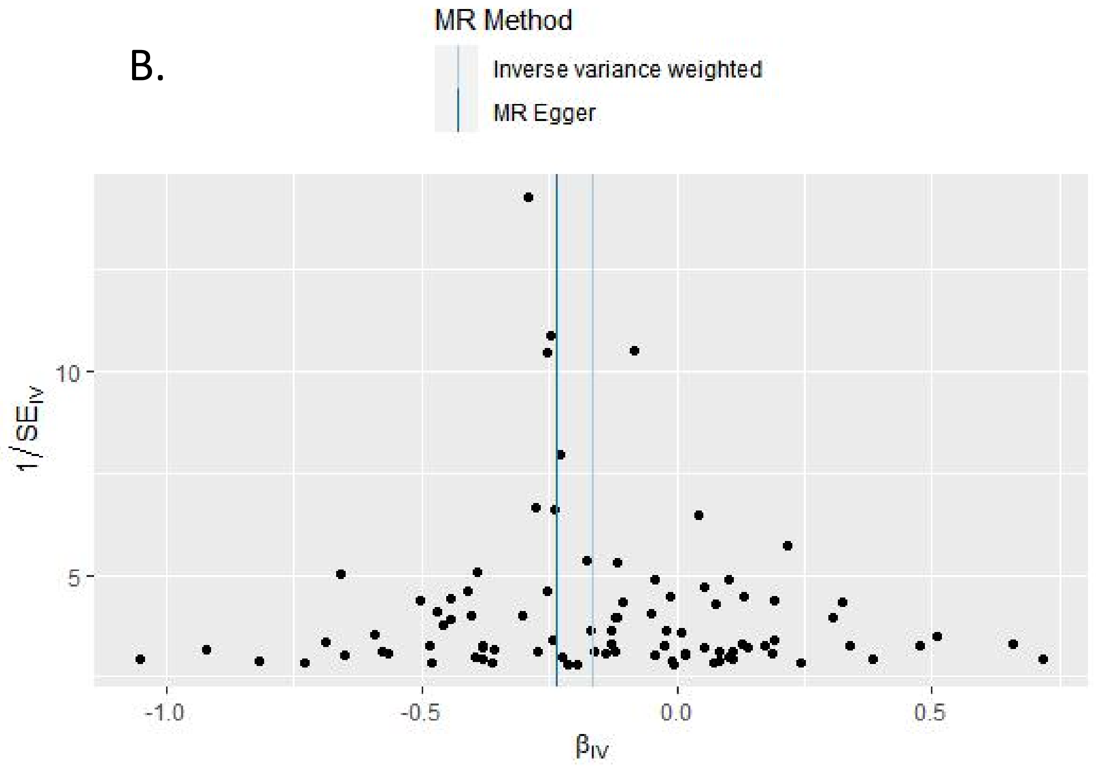

B. Funnel plot for the effect of type 2 diabetes on the risk of bronchial asthma .

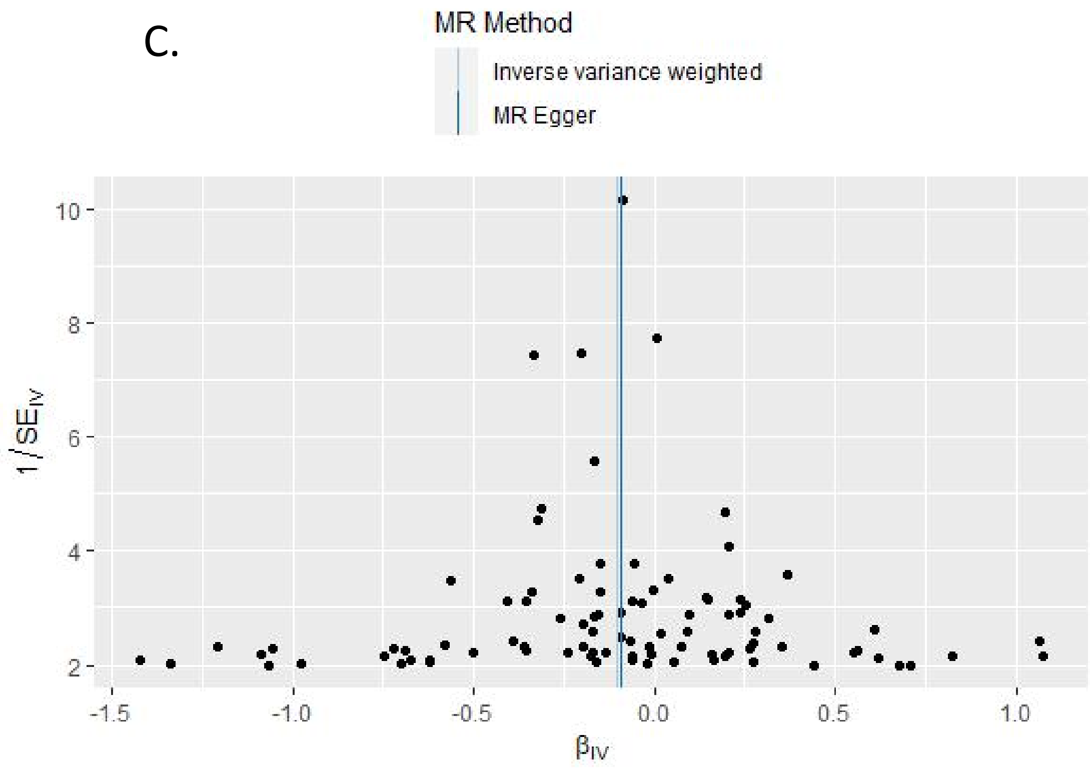

C. Funnel plot for the effect of type 2 diabetes on the risk of lung cancer.

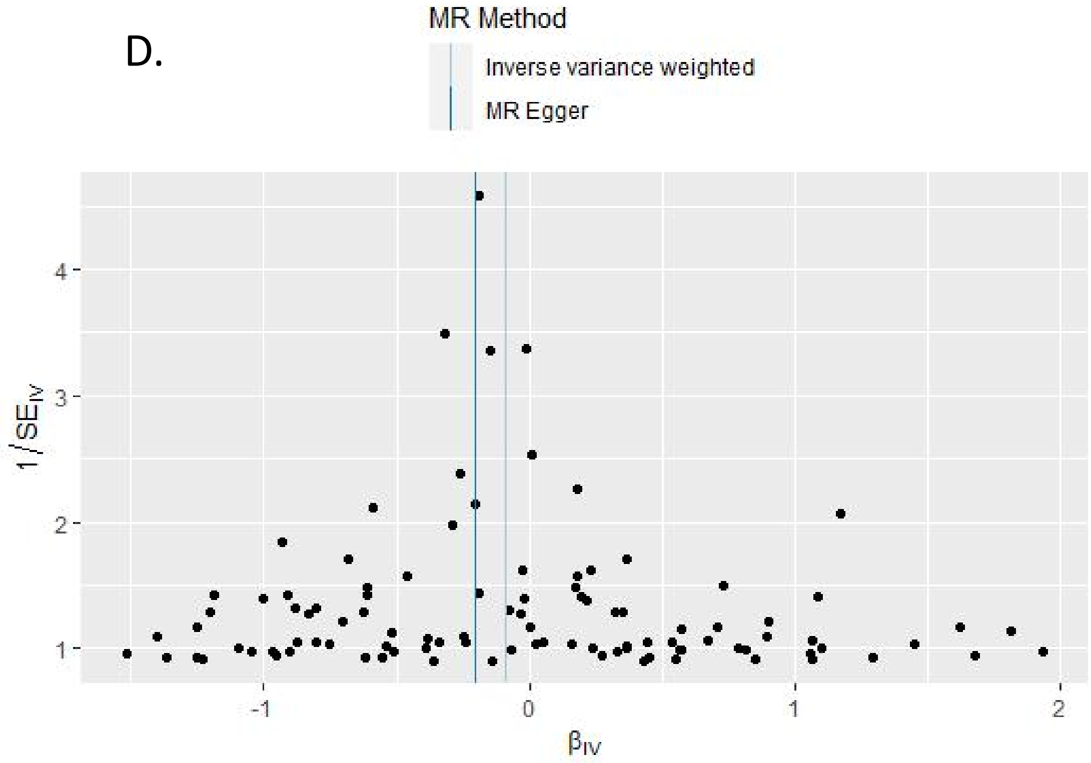

D. Funnel plot for the effect of type 2 diabetes on the risk of interstitial lung disease.

E.

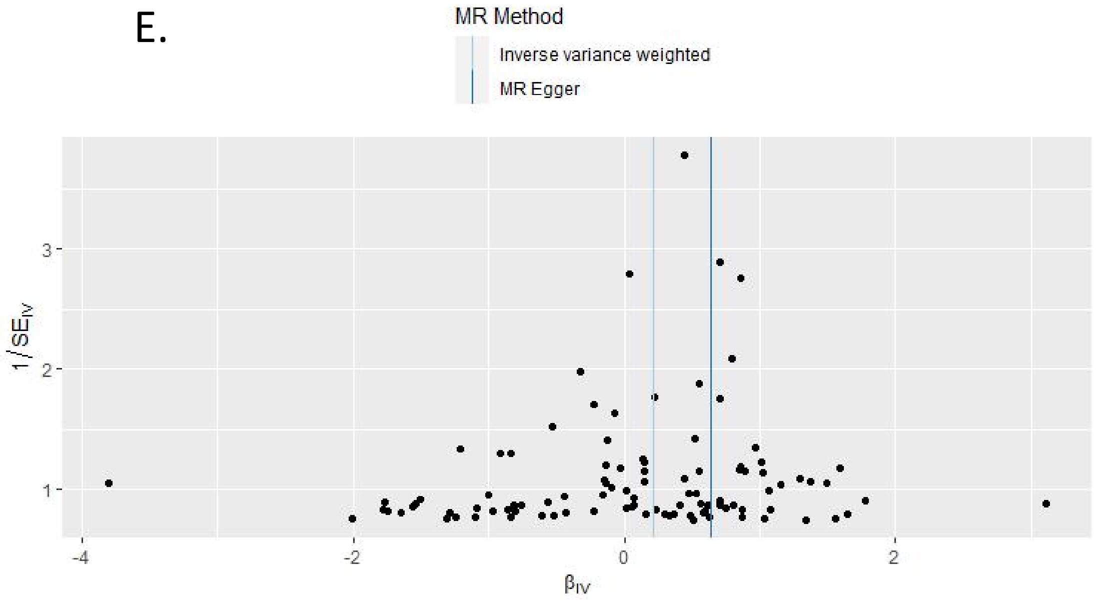

E. Funnel plot for the effect of type 2 diabetes on the risk of pulmonary tuberculosis.
