# Supplementary figures and images for: Causal relationship between type 2 diabetes and common respiratory system diseases: a two-sample Mendelian randomization analysis
Source: Front Med (Lausanne). 2024 Jul 18;11:1332664. doi: 10.3389/fmed.2024.1332664 (PMC11291206; doi:10.3389/fmed.2024.1332664)

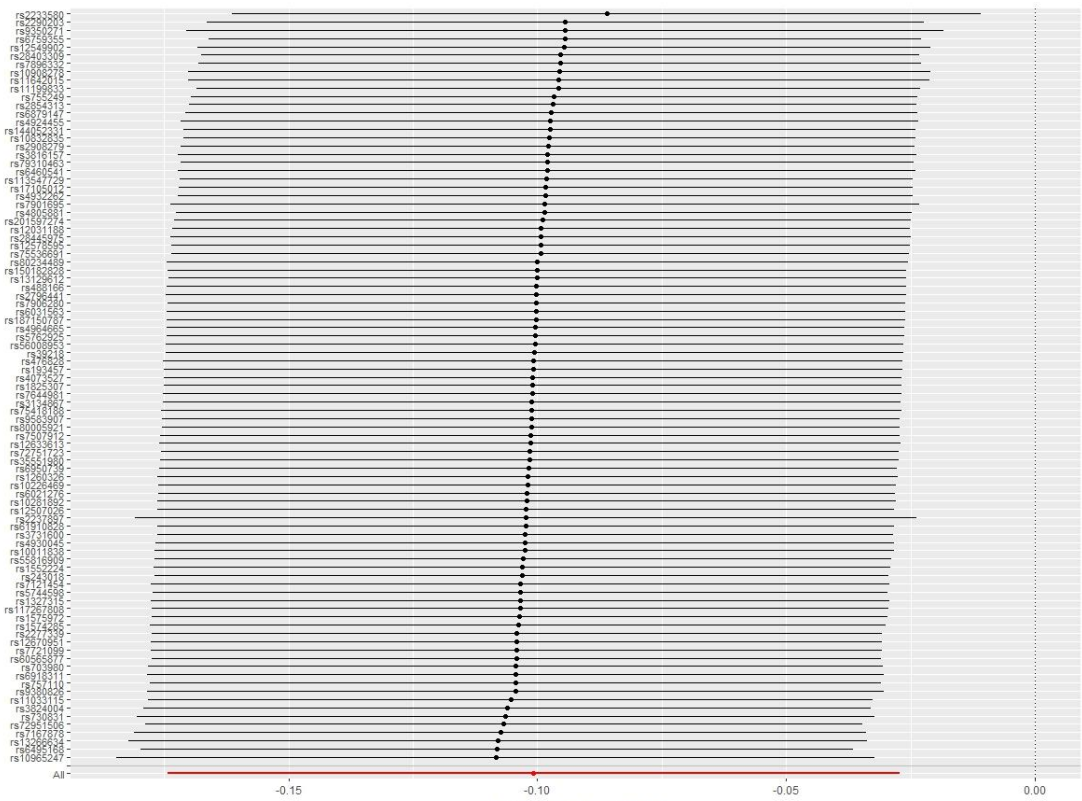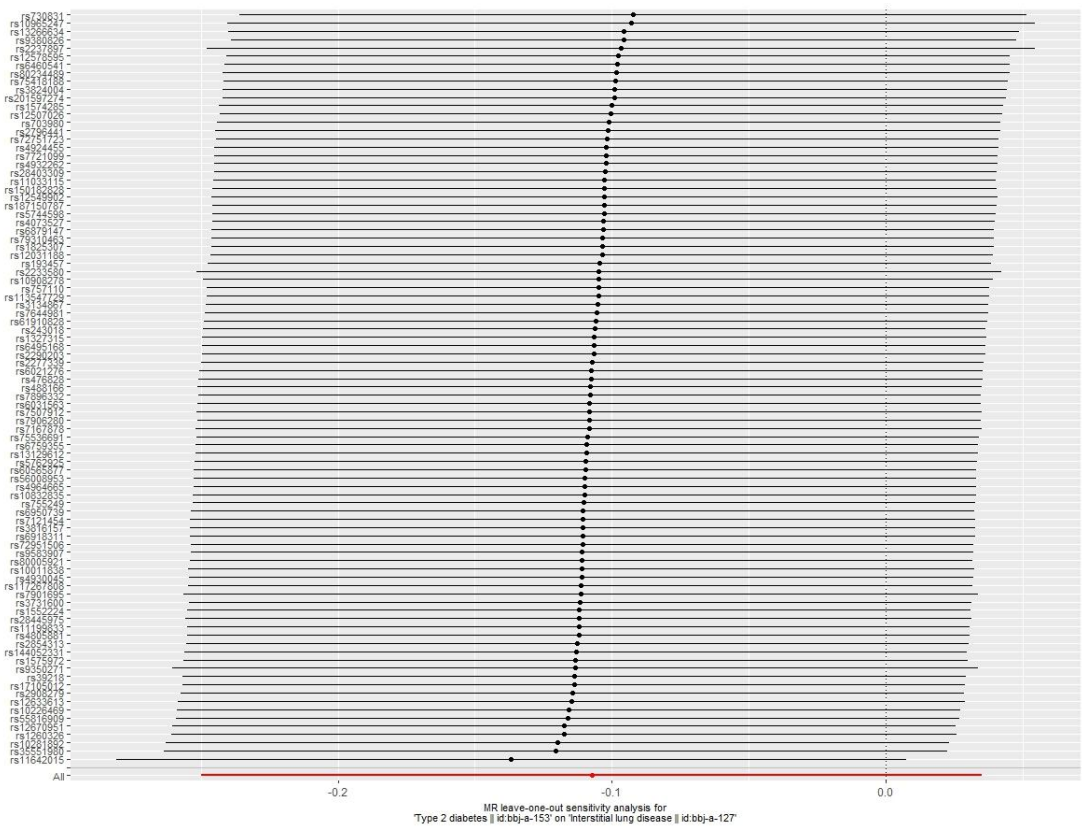

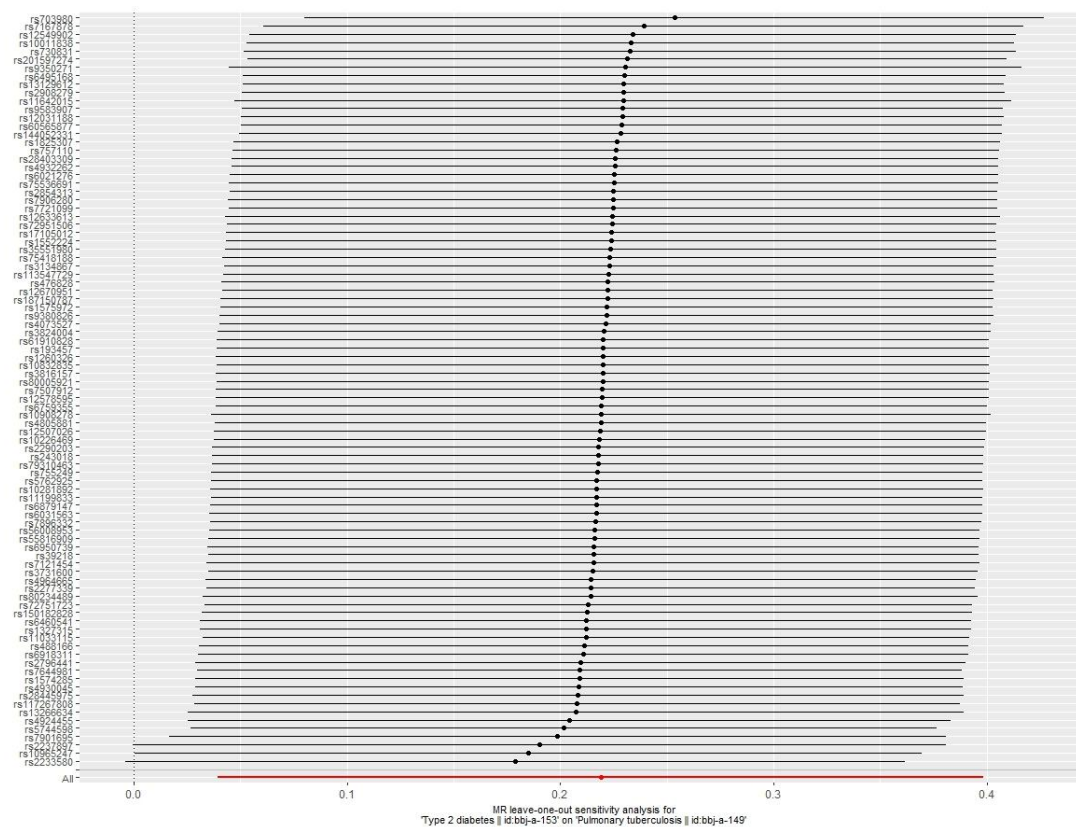

Supplement: Supplementary file 2 [file Data_Sheet_2.PDF]

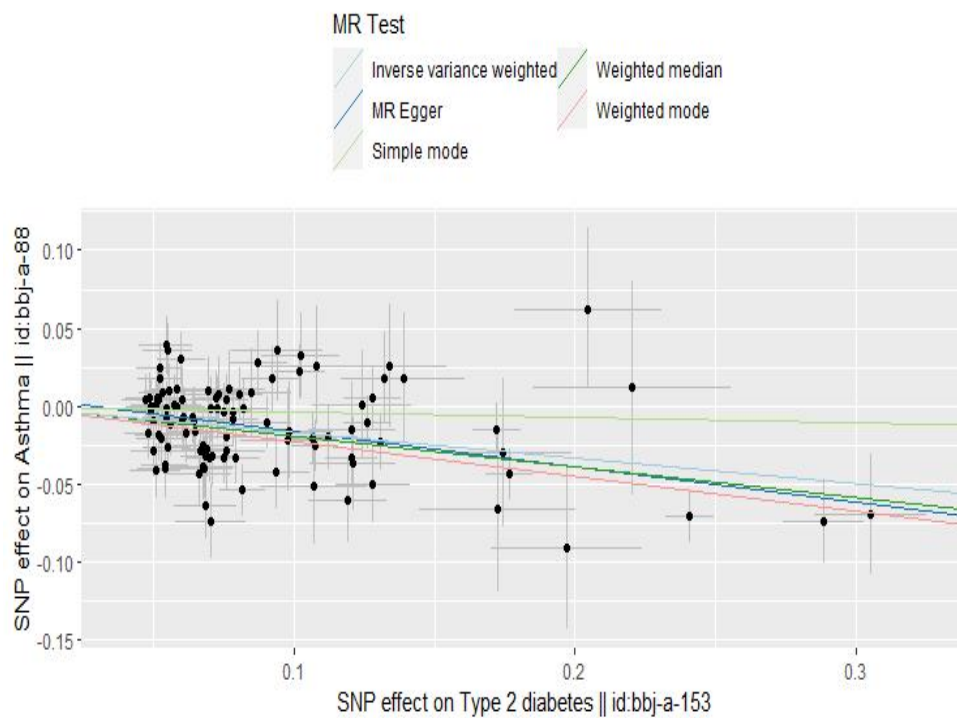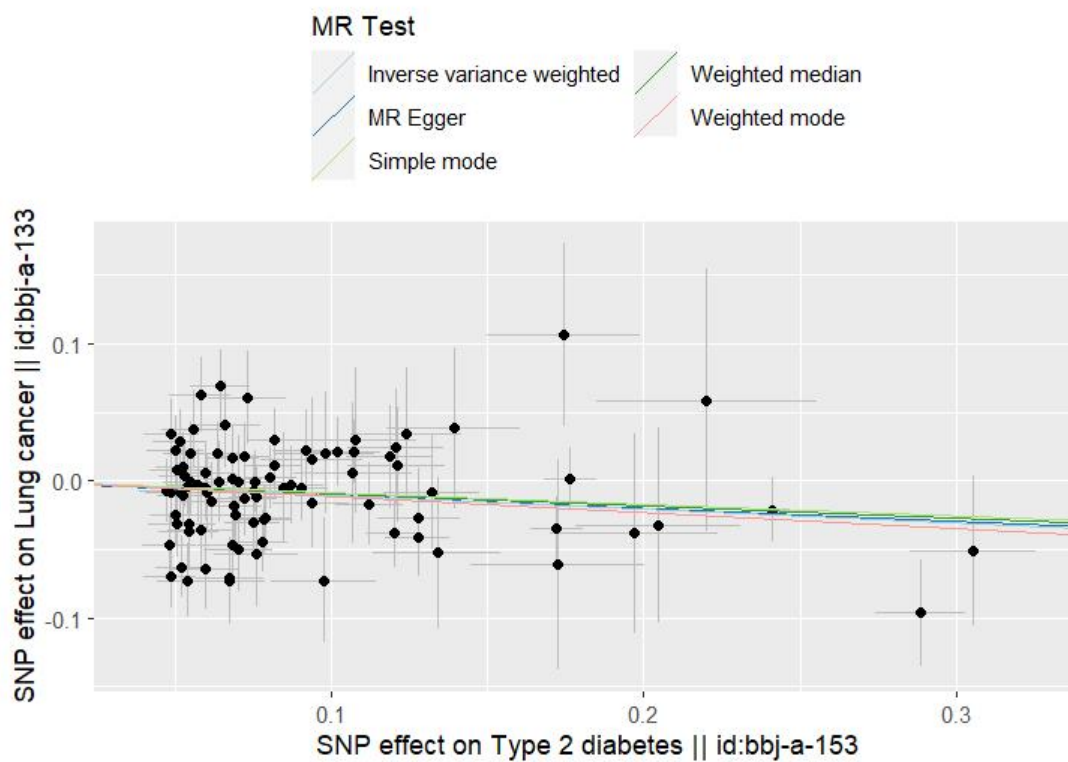

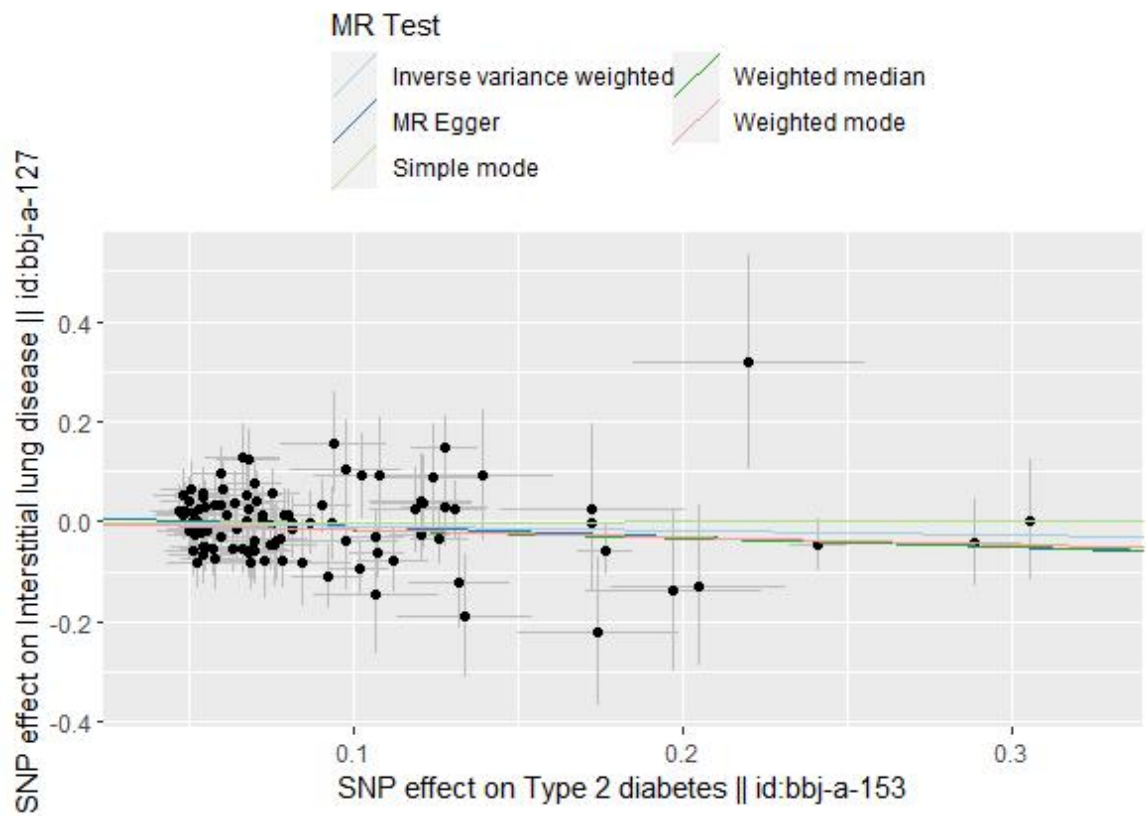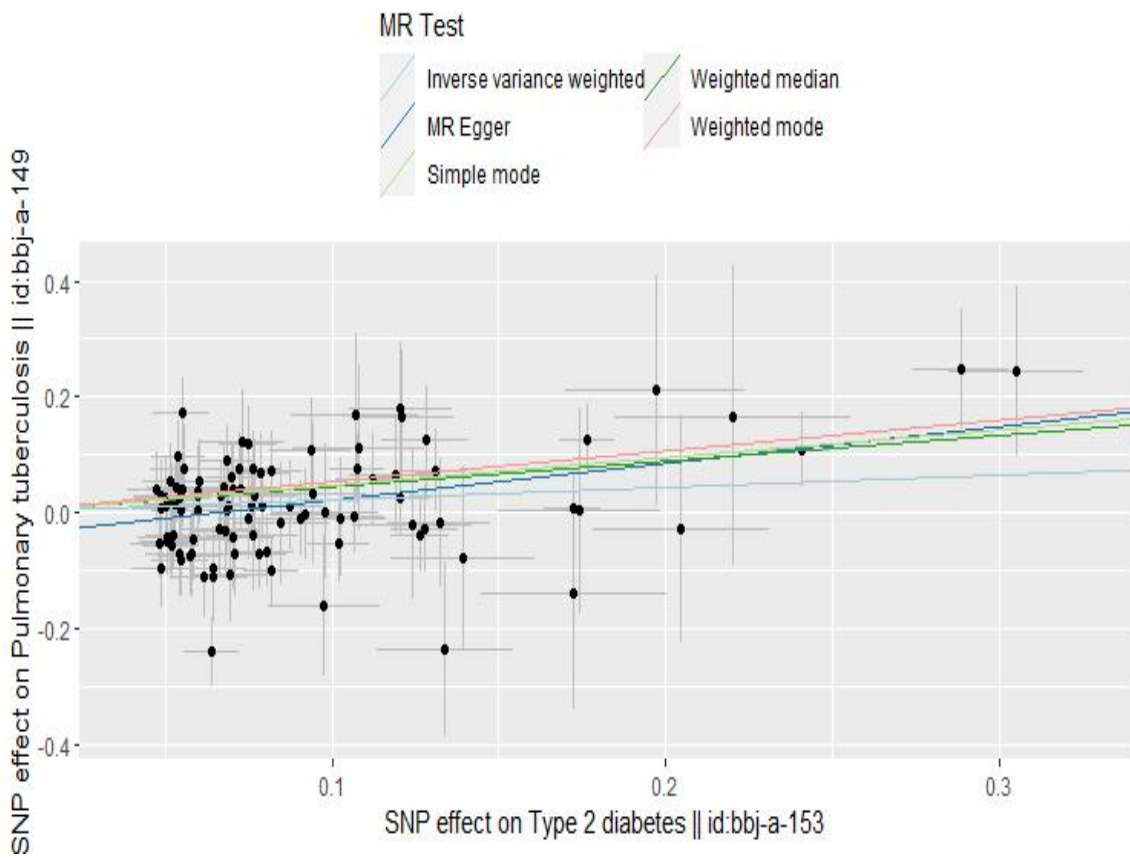

Supplement: Supplementary file 3 [file Data_Sheet_3.PDF]

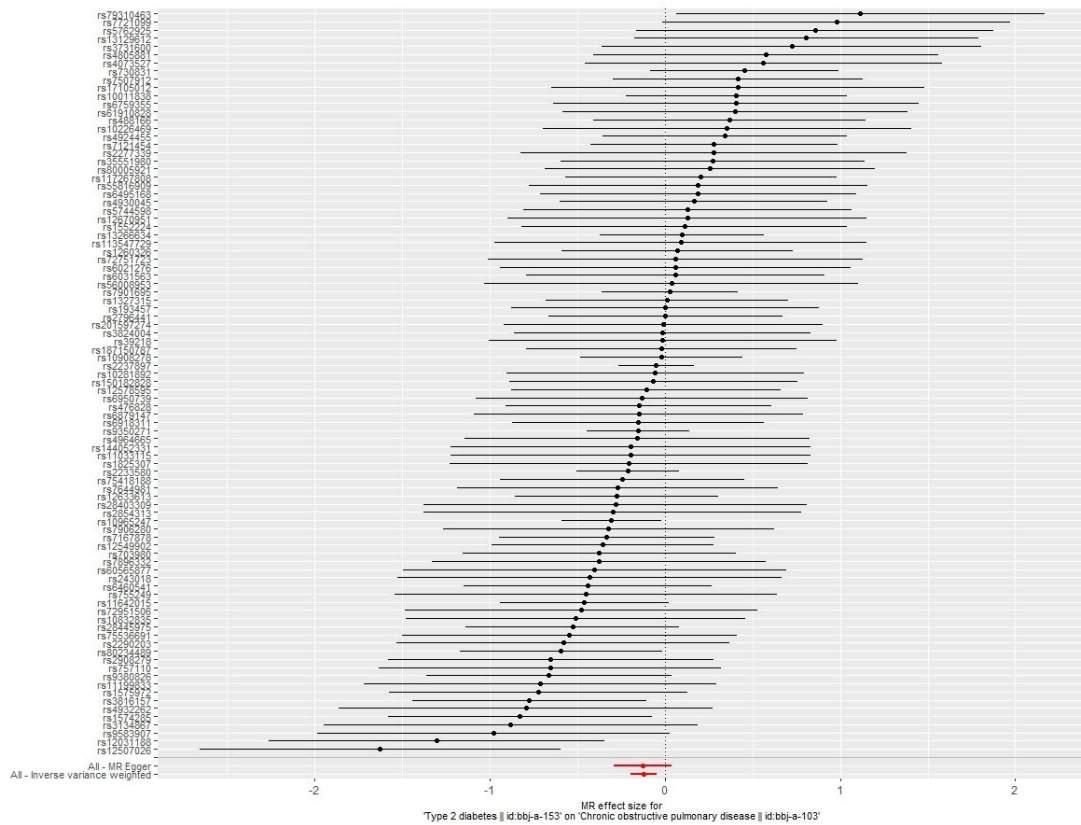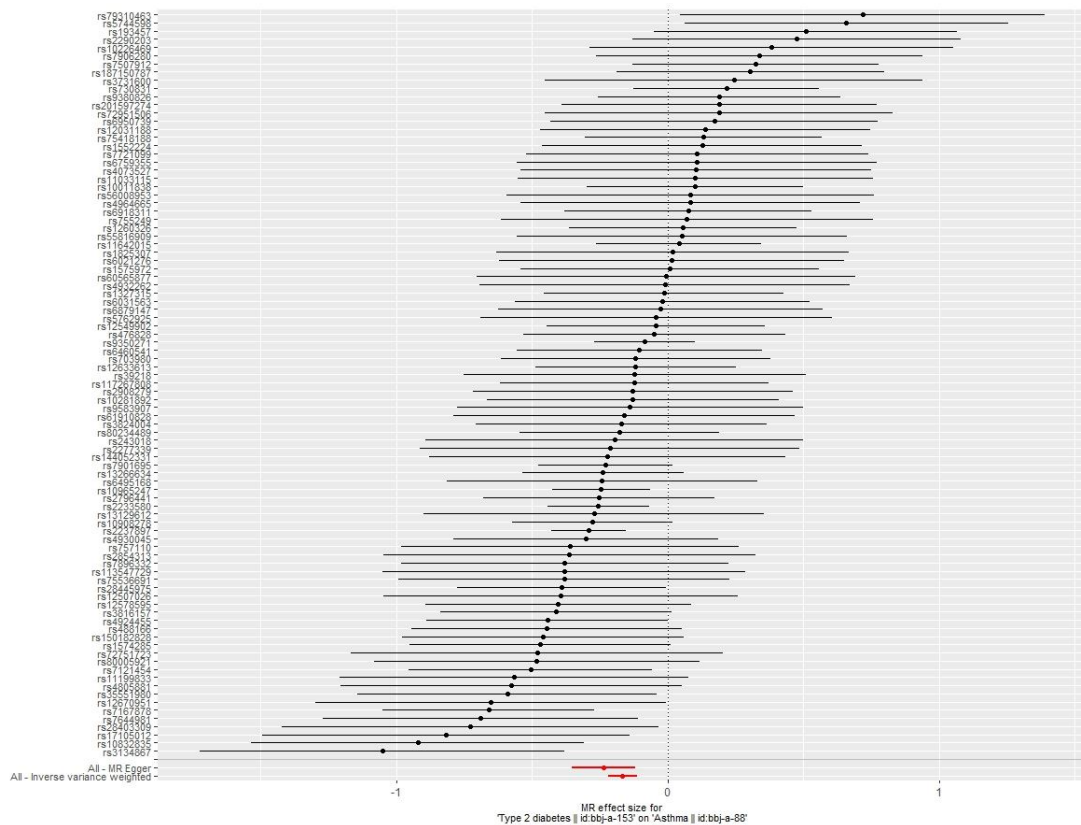



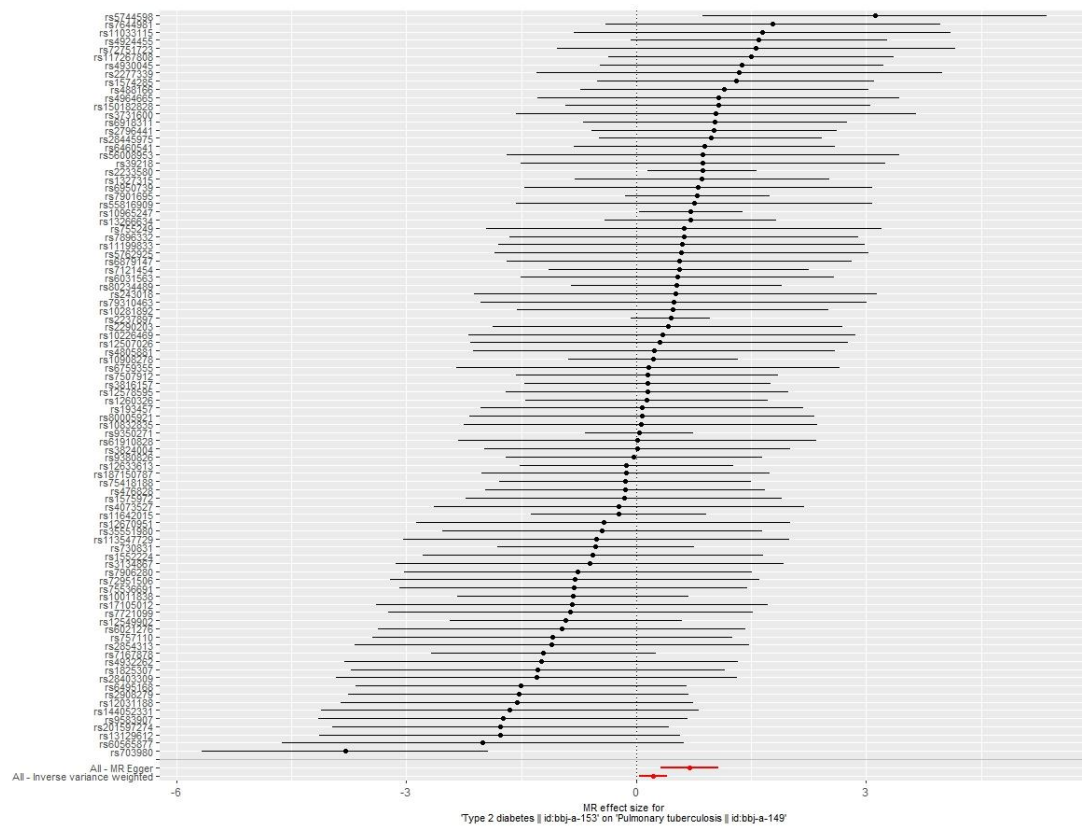

Supplement: Supplementary file 4 [file Data_Sheet_4.PDF]
