# Supplementary material for: Causal relationship between type 2 diabetes and common respiratory system diseases: a two-sample Mendelian randomization analysis
Source: Front Med (Lausanne). 2024 Jul 18;11:1332664. doi: 10.3389/fmed.2024.1332664 (PMC11291206; doi:10.3389/fmed.2024.1332664)
Supplement: Supplementary file 6 [file Table_2.docx]

| **Table S2. Performing Mendelian randomization analysis on recent European population datasets.** | | | | | | | |
| --- | --- | --- | --- | --- | --- | --- | --- |
| **diabetes (exposure)** | | |  | **COPD (outcome)** | | | IVW |
| **dataset** | **Year** | **population** |  | **dataset** | **Year** | **population** |  |
| ebi-a-GCST90093109 | 2022 | European |  | ebi-a-GCST90018807 | 2021 | European | 0.894 |
| finn-b-E4_DM2_STRICT | 2021 | European |  | ebi-a-GCST90018807 | 2021 | European | 0.286 |
| finn-b-E4_DM2NOCOMP | 2021 | European |  | ebi-a-GCST90018807 | 2021 | European | 0.329 |
| ebi-a-GCST90018706 | 2021 | European |  | ebi-a-GCST90018807 | 2021 | European | 0.086 |
| ukb-a-75 | 2019 | European |  | ebi-a-GCST90018807 | 2021 | European | 0.943 |
| ebi-a-GCST90093109 | 2022 | European |  | ukb-b-20464 | 2018 | European | 0.740 |
| finn-b-E4_DM2_STRICT | 2021 | European |  | ukb-b-20464 | 2018 | European | 0.468 |
| finn-b-E4_DM2NOCOMP | 2021 | European |  | ukb-b-20464 | 2018 | European | 0.701 |
| ebi-a-GCST90018706 | 2021 | European |  | ukb-b-20464 | 2018 | European | 0.848 |
| ukb-a-75 | 2019 | European |  | ukb-b-20464 | 2018 | European | 0.821 |

IVW, inverse variance-weighted; T2D, type 2 diabetes. COPD, chronic obstructive plumonary disease.
